# Medical empirical research on forest bathing (*Shinrin-yoku*): a systematic review

**DOI:** 10.1186/s12199-019-0822-8

**Published:** 2019-12-01

**Authors:** Ye Wen, Qi Yan, Yangliu Pan, Xinren Gu, Yuanqiu Liu

**Affiliations:** 1 College of Forestry, Jiangxi Agricultural University, 1101 ZhiMin Road, Nanchang, 330045 China; 2 Jiangxi Academy of Forestry, 1629 FengLin Road, Nanchang, 330032 China

**Keywords:** Forest bathing (*Shinrin*-*yoku*), Systematic review, Methodology

## Abstract

**Aims:**

This study focused on the newest evidence of the relationship between forest environmental exposure and human health and assessed the health efficacy of forest bathing on the human body as well as the methodological quality of a single study, aiming to provide scientific guidance for interdisciplinary integration of forestry and medicine.

**Method:**

Through PubMed, Embase, and Cochrane Library, 210 papers from January 1, 2015, to April 1, 2019, were retrieved, and the final 28 papers meeting the inclusion criteria were included in the study.

**Result:**

The methodological quality of papers included in the study was assessed quantitatively with the Downs and Black checklist. The methodological quality of papers using randomized controlled trials is significantly higher than that of papers using non-randomized controlled trials (*p* < 0.05). Papers included in the study were analyzed qualitatively. The results demonstrated that forest bathing activities might have the following merits: remarkably improving cardiovascular function, hemodynamic indexes, neuroendocrine indexes, metabolic indexes, immunity and inflammatory indexes, antioxidant indexes, and electrophysiological indexes; significantly enhancing people’s emotional state, attitude, and feelings towards things, physical and psychological recovery, and adaptive behaviors; and obvious alleviation of anxiety and depression.

**Conclusion:**

Forest bathing activities may significantly improve people’s physical and psychological health. In the future, medical empirical studies of forest bathing should reinforce basic studies and interdisciplinary exchange to enhance the methodological quality of papers while decreasing the risk of bias, thereby raising the grade of paper evidence.

## Introduction

Subhealth is a third state between health and disease. The most common symptoms of subhealth are fatigue, poor sleep quality, forgetfulness, physical pain, and sore throat, and subhealth also increases the risk of infection and degrades the capacity of the immune system [1, 2]. With the rapid development of the global economy and urbanization, increasing numbers of people have begun to show subhealth symptoms. In a survey conducted by the Ministry of Health, Labor and Welfare of Japan (32,000 Japanese people over 12 years old), 54.2% of respondents considered their stress levels to be “very high” or “relatively high” [3]. It is estimated that one third of American adults have nighttime sleep problems every week, and between 50 and 70 million people complain that nighttime sleep deprivation is mediated by daytime impairment [4, 5]. A meta-analysis showed that between one third and one half of the population in the UK was affected by chronic pain, and the incidence of chronic pain in different body parts among adult residents was 35.0–51.3%, and the incidence of chronic pain increased with age [6]. Under the background of the increasing number of people with subhealth around the world, forest bathing (*Shinrin*-*yoku*) therapy came about, which not only brings people with subhealth a healthy lifestyle advocated by modern medicine but also offers complementary therapies to the sick [7]. The term forest bathing was created in 1982 by the Ministry of Agriculture, Forestry and Fisheries of Japan [8]. It refers to a healing technique that restores the physical and psychological health of the human body through a “five senses experience” (vision, smell, hearing, touch, and taste) when the body is exposed to a forest environment. Forest bathing has positive effects on human physical and mental health [9, 10], especially in enhancing immunity, treating chronic diseases, regulating mood, and reducing anxiety and depression [11–14]. More benefits can be gained from exercising or meditating in a forest environment than in an urban environment [15, 16]. In recent years, although medical empirical research on forest bathing has increased gradually, its healthcare mechanism for the human body has not been clearly defined due to a lack of research results, a low level of evidence, and a disciplinary barrier. Although forestry scholars and medical scholars have carried out relevant research on forest bathing therapy, there are still some limitations due to different research focuses. (1) The theoretical basis of research varies. Medical scholars mainly take evidence-based medicine as the theoretical basis for studying the physiological and psychological stress response of the human body during exposure to the forest environment to demonstrate the health-related effects of forest bathing. Forestry scholars mainly study the health mechanism of forest environmental factors and the relationship among them based on the theory of forestry. (2) The subject of research varies. The research subject of medical scholars is the human body, through studying changes in physiological and psychological indicators to directly verify the health-related effects of forest bathing. The research subject of forestry scholars is the forest environment, through the study of forest environmental factors of different variables to indirectly prove the health benefits of forest bathing. To solve this problem, this study uses the evidence-based medicine system evaluation method to qualitatively integrate the research results. The objectives are as follows: (1) focus on the latest evidence of the relationship between forest environment exposure and human health, (2) assess the methodological quality of individual studies, and (3) provide scientific theoretical guidance for the interdisciplinary integration of forestry and medicine.

## Methods

### Selection criteria

(1) Interventional study on the health effects of forest bathing. (2) Number of intervening measures is less than or equal to 3. (3) Trial was carried out in a forest environment. (4) The study period of the paper was from January 1, 2015, to April 1, 2019. (5) The paper is written in English. (6) Subjects are human.

### Paper search

Through computer retrieval of PubMed, Embase, and Cochrane Library, we screened medical empirical research papers on forest bathing published in the last 5 years, and used a citation traceability method and Google academic search for papers that needed to be supplemented. In this study, the combination of subject words and free words was adopted, and the logical character “OR” was used to link each search term to obtain final search results. Search terms are shown in Table 4 in Appendix.

### Paper screening and data extraction

Paper preliminary screening was conducted independently by one researcher through reviewing titles and abstracts, and data extraction was conducted independently by two researchers. After extraction, cross-checking was conducted, and disputes were resolved through discussion or referring to third-party opinions. Data extraction includes author name, publication year, study design, participant profile, ethical review, sample size, intervention measures, control measures, measurements, and outcomes.

### Quality assessment tool

The methodological quality of the included studies was assessed using the Downs and Black checklist [17], which was used for quantitative evaluation of the quality of papers in randomized controlled trials (RCTs) and non-randomized controlled trials (NRCTs). The evaluation included 27 items from 5 aspects of the paper: reporting, external validity, bias, confounding, and power. The evaluation was carried out by two researchers independently, and any disputes could be resolved through discussion or by referring to the opinions of a third party. The system evaluation report was prepared according to the Preferred Reporting Items for Systematic Reviews and Meta-Analyses [18] declaration standard.

## Results

### Search results

**Fig. 1:**
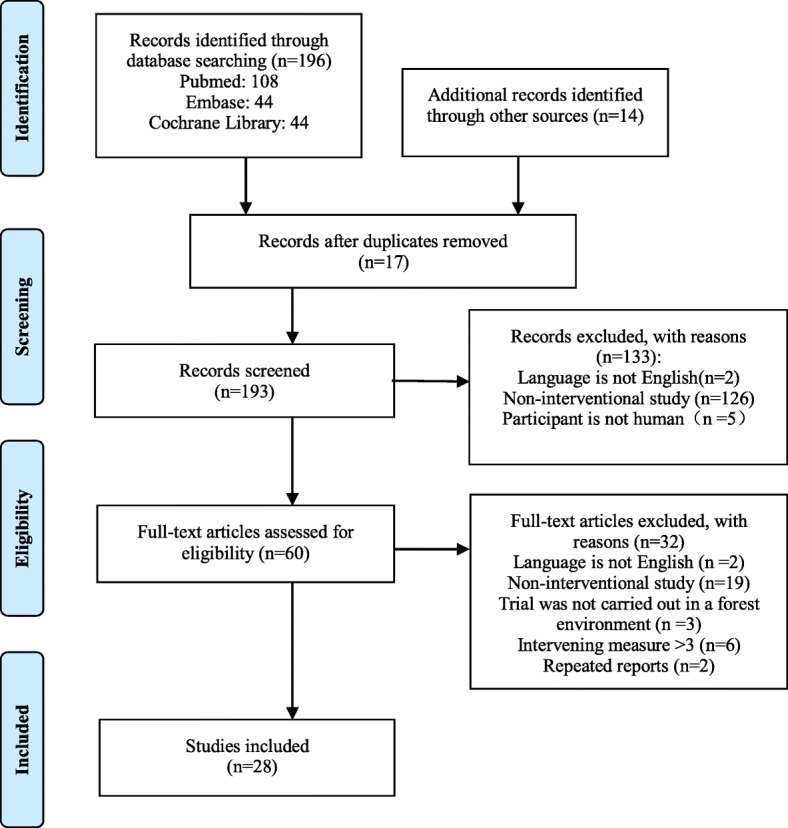
Flow diagram of the screening process

Initially, 210 papers were searched, and 17 duplicate papers and 133 irrelevant papers were removed based on title and abstract. Subsequently, we evaluated the full text and excluded 32 papers. Finally, 28 papers met the criteria for inclusion in the study. The screening process is shown in Fig. 1.

### General characteristics

General characteristics of the included studies are shown in Table 1. A total of five countries or regions, including Japan, South Korea, Poland, China, and Taiwan, have conducted empirical studies on the health effects of forest bathing [19–46], among which 27 studies were conducted in Asian countries [19–34, 36–46] and 1 study [35] in a European country. Japanese scholars had the largest number of studies, publishing 13 papers [19, 20, 22–25, 28, 31, 38, 40, 42–44], accounting for 46% of the total number of included studies, followed by Chinese, South Korean, Taiwanese, and Polish scholars, publishing 6 papers [27, 32, 37, 39, 45, 46], 5 papers [21, 26, 29, 30, 34], 3 papers [33, 36, 41], and 1 paper [35], respectively, accounting for 21%, 18%, 11%, and 4% of the total number of included studies, respectively. Among them, there were 17 RCTs [20, 21, 24–27, 30–32, 35, 37–40, 43, 45, 46], accounting for 61% of the total number of included studies, and 11 NRCTs [19, 22, 23, 28, 29, 33, 34, 36, 41, 42, 44], accounting for 39% of the total number of included studies. The participants were dominated by healthy people, with a total of 17 studies [20, 24, 26, 29, 31, 33–38, 40, 42–46], accounting for 61% of the total number of studies included, and mostly young people aged 18–30. There were 11 studies [19, 21–23, 25, 27, 28, 30, 32, 39, 41] on people with health problems, accounting for 39% of the total number of studies, most of which were middle-aged and elderly people over 45 years old. There were 13 studies [19, 21, 24, 29–31, 33, 34, 37, 38, 40, 43, 45] with more than 50 samples, accounting for 46% of the total number of included studies, 8 studies [20, 22, 23, 25, 27, 36, 41, 44] with less than 20 samples and 7 studies [26, 28, 32, 35, 39, 42, 46] with 20–50 samples, accounting for 29% and 25% of the total number of included studies, respectively. There were 20 forest bathing studies [19, 20, 22–26, 28, 31, 33, 35–38, 40, 42–46] that lasted for 1–3 days, accounting for 71% of the total number of included studies. There were 8 forest bathing studies [21, 27, 29, 30, 32, 34, 39, 41] that lasted for more than 3 days, accounting for 29% of the total number of included studies. Most scholars have taken ethical considerations into account when carrying out research. A total of 25 studies [19, 20, 22–44] have passed the ethical review, accounting for 89% of the total included studies. This was not mentioned in 3 studies [21, 45, 46], accounting for 11% of the total number of included studies. There were 3 interdisciplinary studies [44–46], accounting for 11% of the total included studies.

**Table 1:** General characteristics of included studies (*n* = 28)

Characteristic	Categories	No. (%)
Country or region	China	6 (21)
	Korea	5 (18)
	Japan	13 (46)
	Poland	1 (4)
	Taiwan	3 (11)
Research design	RCT	17 (61)
	NRCT	11 (39)
Participant	Healthy people	17 (61)
	People with health problems	11 (39)
Average age (years)	18 ≤	1 (3.5)
	> 18 ≤ 30	12 (43)
	> 30 ≤ 45	1 (3.5)
	> 45	12 (43)
	Age unknown	2 (7)
Sample size	≤ 20	8 (29)
	> 20 ≤ 50	7 (25)
	> 50	13 (46)
Time	3 days ≤	20 (71)
	> 3 days	8 (29)
Ethical consideration	Yes	25 (89)
	No	3 (11)
Interdisciplinary research	Yes	3 (11)
	No	25 (89)

### Intervention measures and control measures

The detailed characteristics of the included research papers are shown in Tables 2 and 3. All studies take forest or urban environment exposure as the trial premise, and more than one or two intervention measures are adopted to carry out the trial, and some control measures are imposed. The interventions are mostly walking, meditation, yoga, Pilates, sightseeing, and crafts. The “five senses experience” and exercise are at the core. The control measures of each study are similar, mainly including the following: (1) control trial time and activity space; (2) prohibit or control tobacco, alcohol, and caffeine intake; (3) prohibit or allow use of drugs and electronic products; (4) control of accommodation and diet; (5) consideration of female physiological period factors; and (6) increase buffer time (many hours or days) in a cross-over study to prevent carryover effect.

**Table 2:** Medical empirical research (*n* = 25)

Authors (year)	Research design	Participants		Intervention measures	Control measures	Measurements and outcomes	
		Trial group	Control group			Self-report measures	Physiological measures
Horiuchi (2015) [19]	NRCT (before-after study) ※	1) Response group: Male and female participants, average age was 63.9 years (*n* = 27). 2) Non-response group: Male and female participants, average age was 61.6 years (*n* = 27).	N/A	Participants were exposed to forest environment and the activity was carried out for 90 min	1) Participants were divided into 2 groups according to the changes of mean arterial pressure before and after forest bathing (>5% was the response group, <5% was the non-response group). 2) Some participants were given medications for hypertension, diabetes, hyperlipidemia, hyperuricemia, and osteoporosis. 3) Smoking and caffeine were banned 12 h before the trial, and alcohol was banned 24 h before the trial	1) Response group POMS: D*↓ V*↑ T-A*↓ F*↓ C*↓ A-H*↓ 2) Non-response group POMS: D*↓ V*↑ T-A*↓ F*↓ C*↓ A-H↓	1) Response group: SBP*↓ DBP*↓ Mean arterial pressure*↓ Salivary amylase↓ 2) Non-response group: SBP*↓ DBP↓ Mean arterial pressure*↓ Salivary amylase↑
Igarashi (2015) [20]	RCT (cross-over study) ※	Female participants, average age was 46.1 years (*n* = 4 or 1)	Female participants, average age was 46.1 years (*n* = 4 or 1)	After a 3-min rest, the participants sat and watched the kiwi orchard for 10 min (or the building site); after a 3-min rest, the participants sat and watched the building site (or the kiwi orchard), each group was asked to view 2 trial sites	1) The trial began in the summer. 2) Seventeen participants were divided into five groups. 3) Participants avoided menstruation and did not drink or smoke. 4) Lived in the suburbs. 5) The two trial sites are close to each other	SD method: Comfortable feeling^#^↑ Natural feeling^#^↑ Relaxed feeling^#^↑ POMS: D^#^↓ V^#^↑ T-A^#^↓ F^#^↓ C^#^↓ A-H ^#^↓	lnHF #↑ lnLF/lnHF↓ Heart rate↓
Kang (2015) [21]	RCT	Male and female participants, average age was 54.8 years (*n* = 32)	Male and female participants, average age was 50 years (*n* = 32)	In the morning, the trial group and control group were exposed to the forest environment and walked for 2 h In the afternoon, the trial group performed additional stretching and intensive exercises for 4 h	1) The trial began in late spring and lasted five days. 2) Participants selection criteria: Adults over 20 years of age with posterior neck pain for more than 3 months, and VAS grades over 4	VAS: VAS on the first day*↓ VAS on the end day*↓ Cervical range of motion*↑ Neck disability index*↓ EuroQol 5D-3 L VAS*↑ EuroQol 5D-3 L index*↑ McGill pain questionnaire*↓ Trigger points in the posterior neck region^#^↓	N/A
Ochiai (2015a) [22]	NRCT (before-after study) ※	Male participants with high normal blood pressure, age range 40–72 years (*n* = 9)	N/A	On trial day, participants were exposed to forest environment for activities and rest from 10:30 to 15:05	1) The trial was carried out in early autumn, and the average air temperature was 21.5 °C. 2) No alcohol or conversation was allowed during the trial, and cell phones were allowed only during breaks	SD method: Comfortable feeling↑ Natural feeling*↑ Relaxed feeling*↑ POMS: D↓ V↓ T-A*↓ F↓ C*↓ A-H*↓ POMS total mood disturbance*↓	SBP*↓ DBP*↓ Urinary adrenaline levels *↓ Serum cortisol levels *↓
Ochiai (2015b) [23]	NRCT (before-after study) ※	Female participants, the average age was 62.2 years (*n* = 17)	N/A	On trial day, participants were exposed to forest environment for activities and rest from 10:32 to 15:13	1) The trial was carried out in summer, and the average air temperature was 21.5 °C. 2) Except for 6 participants who were taking medication to control their blood pressure, the rest of the participants had no other physical or psychological diseases. 3) No alcohol or cell phones were allowed during the trial	SD method: Comfortable feeling*↑ Natural feeling*↑ Relaxed feeling*↑ POMS: T-A*↓ F↓ V*↑	Pulse rate*↓ Salivary cortisol concentration *↓
Song (2015a) [24]	RCT (cross-over study) ※	Male participants, the average age was 21.5 years (*n* = 6)	Male participants, the average age was 21.5 years(*n* = 6)	Day 1, the trial group was exposed to forest environment and walked for 15 min, while the control group was exposed to urban environment and walked for 15 min. Day 2, the two groups interchanged environments	1) The trial lasted for 2 days. 2) Smoking and drinking are prohibited during the trial. 3) The trial was conducted several times and a total of 92 participants participated	N/A	If the participants had high initial blood pressure and pulse, forest walking could reduce these two indicators. The results were reversed if the participants had lower initial blood pressure and pulse
Song (2015b) [25]	RCT (cross-over study) ※	Male participants with hypertension or high normal blood pressure, the average age was 58 years (*n* = 10)	Male participants with hypertension or high normal blood pressure, the average age was 58 years(*n* = 10)	Day 1, the trial group was exposed to forest environment and walked for 17 min, while the control group was exposed to urban environment and walked for 17 min. Day 2, the two groups interchanged environments	1) The trial lasted for 2 days. 2) When the trial was carried out, the average air temperature in the forest was 21.4 °C, and that in the city was 28.1 °C. 3) Smoking, alcohol and caffeine consumption were prohibited during the trial. 4) Participants who were on medication were excluded. 5) Trial at the same time every day	SD method: Comfortable feeling^#^↑ Natural feeling^#^↑ Relaxed feeling^#^↑ POMS: D^#^↓ V^#^↑ T-A^#^↓ F^#^↓ C^#^↓ A-H^#^↓	lnHF ^#^↑ Pulse rate^#^↓
Im (2016) [26]	RCT (cross-over study) ※+	Male and female participants, age range 18–35 years (*n* = 19)	Male and female participants, the age range 18–35 years(*n* = 22)	In the morning, the trial group was exposed to forest environment for 2 h, while the control group was exposed to urban environment for 2 h. In the afternoon, the two groups interchanged environments	1) The trial began in the summer. 2) The participants had no mental illness, allergic rhinitis or bronchitis. 3) Bachelor’s degree or above and live in city. 4) To avoid carryover effect, the interval between morning trial and afternoon trial was 2 h. 5) Alcohol consumption was restricted 12 h before the test, and food consumption was restricted 1 h before the test. Smoking and drinking were prohibited during the test, and electronic products were restricted. 6) All groups had the same diet	Stress response inventory: Total^#^↓ Somatic symptoms^#^↓ Depressive symptoms^#^↓ Anger symptoms↓	IL-6↑ IL-8^#^↓ TNF-α^#^↓ GPx^#^↑
Jia (2016) [27]	RCT※	Male and female participants with COPD (*n* = 10)	Male and female participants with COPD (*n* = 8)	In the morning, the trial group was exposed to forest environment and walked for 90 min, while the control group was exposed to urban environment and walked for 90 min. Afternoon is the same as morning	1) The trial began in the summer. 2) The participants did not have acute exacerbation. 3) Participants have the same accommodation and schedule. 4) The trial lasted for 4 days	POMS: D*↓ V↑ T-A*^#^↓ F↓ C↓ A-H*↓	NK cells *↓ CD8+ T-lymphocytes expressing perforin *^#^↓ NKT-like cells *^#^↓ IL-6*^#^↓ IL-8*^#^↓ Interferon-γ*^#^↓ TNF-α↓ IL-1β^#^↓ CRP^#^↓ Pulmonary and activation-regulated chemokine*^#^↓ Tissue inhibitor of metalloproteinase-1*^#^↓ Surfactant protein D ^#^↓ Cortisol^#^↓ Epinephrine*^#^↓
Li (2016) [28]	NRCT※	Male participants with hypertension or high normal blood pressure, age range 40–69 years (*n* = 19)	Male participants with hypertension or high normal blood pressure, age range 40–69 years (*n* = 19)	In the first trial, the control group was exposed to urban environment and walked 2.6 km. In the second trial, the control group was exposed to forest environment and walked 2.6 km	1) The trial began in the summer. 2) Participants did not take any antihypertensive drugs. 3) No alcohol was allowed and the diet was the same during the trial. 4) The interval between the two trials was one week	POMS: D^#^↓ V^#^↑ T-A^#^↓ F^#^↓ C^#^↓ A-H!	SBP! DBP! Pulse rate ^#^↓ Triglycerides! Cho! LDL-Cho! HDL-Cho! Remnant-like particles Cho! Adiponectin^#^↑ Glycated hemoglobin! Blood glucose! Insulin! Dehydroepiandrosterone sulfate! CRP! Epinephrine↓ Norepinephrine *^#^↓ Dopamine^#^↓
Bang (2017) [29]	NRCT※	Male and female participants, the average age was 24.8 years (*n* = 51)	Male and female participants, the average age was 23.8 years (*n* = 48)	The participants walked in the campus forest once a week for 40 min	1) The trial began in the autumn. 2) The trial lasted for 6 weeks. 3) The trial group received extra messages of encouragement during the trial and attended a stress management seminar	Health-promoting Lifestyle profile II: Total^#^↑ Responsibility for health^#^↑ Physical activity↑ Healthy nutrition↑ Social relations↑ Stress management^#^↑ Spiritual growth↑ BDI score^#^↓	SBP #↑ DBP↑ Cho↓ HDL-Cho↓ LDL-Cho↑ Triglycerides^#^↓ Bone density^#^↑ Body Mass Index↑ Percent of body fat↑ lnLF/lnHF↑ Parasympathetic nerve activity↑
Chun (2017) [30]	RCT※+	Male and female participants with chronic stroke, the average age was 60.8 years (*n* = 30)	Male and female participants with chronic stroke, the average age was 60.8 years (*n* = 29)	The trial group was exposed to forest environment for meditation and walking. The control group was exposed to urban environment for meditating and walking	2) The trial lasted for 4 days	BDI score*^#^↓ Score of 17-item version of the Hamilton Depression Rating Scale*^#^↓ STAI score*^#^↓	Reactive oxygen metabolites↓ Biological antioxidant potential*^#^↑
Kobayashi (2017) [31]	RCT (cross-over study)※	Male participants, the average age was 21.7 years (*n* = 12)	Male participants, the average age was 21.7 years (*n* = 12)	Day 1, the trial group was exposed to forest environment, while the control group was exposed to urban environment. Day 2, the two groups interchanged environments	1) The trial began in the summer and early autumn. 2) The trial lasted for 2 days. 3) 34 forests and cities were selected for the trial, and a total of 34 trials were carried out	N/A	Salivary cortisol concentration^#^↓
Mao (2017) [32]	RCT※	Male and female participants with chronic heart failure, age range 65–80 years (*n* = 23)	Male and female participants with chronic heart failure, age range 65–80 years (*n* = 10)	The trial group and control group were exposed to forest and urban environment, respectively, and walked for 1.5 h in the morning and afternoon	1) The trial began in the summer. 2) The trial lasted for 5 days. 3) All groups had the same diet. 4) Smoking, drinking alcohol and caffeinated beverages were prohibited during the trial. 5) Medication taken normally during the trial	POMS: D*^#^↓ V↓ T-A^#^↓ F↓ C*↓ A-H*↓	BNP*^#^↓ N-terminal pro-BNP! Endothelin-1^#^↓ ANGII↓ ANGII receptor type 1↑ ANGII receptor type 2 *↑ Angiotensinogen↓ IL-6^#^↓ TNF-α↓ CRP↓ Total superoxide dismutase^#^↑ Malondialdehyde^#^↓
Yu (2017) [33]	NRCT (before-after study)※	Male and female participants, age range 45–86 years (*n* = 128)	N/A	participants were recruited at the gate of the forest park to conduct a 2-h forest tour and walk a total of 2.5 km	1) The trial began in the summer. 2) Smoking, drinking alcohol and caffeinated beverages were prohibited during the trial	POMS: D*↓ V*↑ T-A*↓ F*↓ C*↓ A-H*↓ STAI score*↓	Pulse rate *↓ SBP *↓ DBP*↓ lnHF↓ lnLF/lnHF↑
Bang (2018) [34]	NRCT (before-after study)※	Elementary school students, the average age was 11.83 years (*n* = 24)	Elementary school students, the average age was 11.75 years (*n* = 28)	The trial group allocated 30 min for the lecture and 60 min for the forest activities, while the control group took only indoor classes	1) The trial began in the summer. 2) Once a week for 10 weeks. 3) Children with medical treatment and contraindications to exercise were excluded	1) Trial group Health status questionnaire: Perceived health status↑ Rosenberg Self-Esteem Scale: Self-esteem*↑ Children’s Depression Inventory: D*↓ Peer relationship instrument: Peer relationships↓ Conners-Wells Adolescents Self-Report Scales: Attention deficit and hyperactivity↑ 2) Control group Health status questionnaire: Perceived health status↑ Rosenberg Self-Esteem Scale: Self-esteem↓ Children’s Depression Inventory: D↓ Peer relationship instrument: Peer relationships↑ Conners–Wells Adolescents Self-Report Scales: Attention deficit and hyperactivity↓	1) Trial group lnLF/lnHF↑ 2) Control group lnLF/lnHF↓
Bielinis (2018) [35]	RCT※	Male (*n* = 18) and female (*n* = 13) participants, the average age was 21.45 years	Male (*n* = 18) and female (*n* = 13) participants, the average age was 21.45 years	The trial group was exposed to the forest environment (deciduous broad-leaved forest) and watched the scenery for 15 min, while the control group was exposed to the urban environment and watched the scenery for 15 min	1) The trial began in the winter. 2) No talking with each other during the trial	Positive and negative affect schedule: Positive*^#^↑ Negative^#^↓ POMS: D^#^↓ V*^#^↑ T-A^#^↓ F*^#^↓ C^#^↓ A-H^#^↓ Restorative Outcome Scale scores*^#^↑ Subjective Vitality Scale scores*^#^↑	N/A
Chen (2018) [36]	NRCT (before-after study)※	Female participants, age range 36–62 years (*n* = 16)	N/A	Day 1, participants were exposed to forest environments for walking. Day 2, participants were exposed to forest environments and made handicrafts	1) The average air temperature during the trial was 13.8 °C. 2) participants had the same accommodation and diet. 3) Smoking and stimulant foods were prohibited during the trial	POMS: D↓ V*↑ T-A*↓ F*↓ C*↓ A-H*↓ STAI scores*↓	Pulse rate↓ SBP*↓ DBP↓ Salivary α-amylase↓
Hassan (2018) [37]	RCT (cross-over study) ※	Male and female participants, age range 19–24 years (*n* = 30)	Male and female participants, age range 19–24 years (*n* = 30)	Day 1, the trial group was exposed to forest environment and walked for 15 min, while the control group was exposed to urban environment and walked for 15 min. Day 2, the two groups interchanged environments	1) The average air temperature on the first day was 22 °C, and the average air temperature on the second day was 27 °C. 2) The trial lasted for 2 days. 3) Participants had the same accommodation and diet	STAI scores^#^↓ SD method: Comfortable feeling^#^↑ Natural feeling^#^↑ Relaxed feeling^#^↑	SBP^#^↓ DBP^#^↓ High alpha brain waves^#^↑ High beta brain waves^#^↑ Relaxation scores^#^↑ Attention scores^#^↑
Kobayashi (2018) [38]	RCT (cross-over study) ※	Male and female participants, age range 19–29 years (*n* = N/A)	Male and female participants, age range 19–29 years (*n* = N/A)	Day 1, the trial group was exposed to forest environment and walked for 15 min, while the control group was exposed to urban environment and walked for 15 min. Day 2, the two groups interchanged environments	1) The trial was carried out in 57 cities and forest areas. 2) The trial lasted for 2 days. 3) The total number of participants was 684, and the numbers of participants from trial group or control group were different in every trial	N/A	lnHF^#^↑ lnLF/lnHF ^#^↓
Mao (2018) [39]	RCT※	First trial, male and female participants with chronic heart failure (*n* = 23). Second trial, male and female participants with chronic heart failure (*n* = 10)	Male and female participants with chronic heart failure (*n* = 10)	The trial group was exposed to forest environment, while the control group was exposed to urban environment	1) The trial was carried out twice, the first time in late summer for 5 days, and the second time in early autumn for 5 days. 2) No alcohol or tea was allowed during the trial	N/A	BNP*^#^↓ IL-6! TNF-α*^#^↓ Total superoxide dismutase! Malondialdehyde^#^↓ SBP↓ DBP↓
Song (2018) [40]	RCT (cross-over study) ※	Male participants, average age was 21.7 years (*n* = 6)	Male participants, the average age was 21.7 years (*n* = 6)	Day 1, the trial group was exposed to forest environment, while the control group was exposed to urban environment. Day 2, the two groups interchanged environments	1) The trial was conducted in the summer from 2005 to 2013 and lasted for 2 days at a time. 2) The study was conducted in 52 urban and forest areas with a total of 585 participants. 3) Smoking and drinking alcohol were prohibited, and limited caffeine intake	POMS: D^#^↓ V^#^↑ T-A^#^↓ F^#^↓ C^#^↓ A-H^#^↓	N/A
Tsao (2018) [41]	NRCT (before-after study)※	Male and female participants, the average age was 60.4 years (*n* = 11)	N/A	Participants were exposed to forest environment and walked 1.5 h in the morning and afternoon (in two different forests)	1) The trial began in the winter. 2) The trial lasted for 5 days. 3) The participants had no diabetes, cardiovascular disease or other major diseases. 4) Diet control began 10 days before the trial	N/A	NK cells↑ NK cells activity*↑
Wang (2018) [42]	NRCT (before-after study) ※	Male and female college students (*n* = 22)	N/A	The participants carried out a 2 to 3-day forest trip	1) The trial was conducted in the fall of 2015, 2016 and 2017. 2) Participants had the same diet. 3) Smoking, coffee and tea were not allowed during the trial	N/A	1) Day after the trial: Urinary hydrogen peroxide*↓ Urinary 8-hydroxy-2’deoxyguanosine*↓ 2) One week after the test: Urinary hydrogen peroxide*↓ Urinary↓ 8-hydroxy-2’deoxyguanosine*↓
Song (2019) [43]	RCT (cross-over study) ※	Female participants, the average age was 21 years (*n* = 6)	Female participants, the average age was 21 years (*n* = 6)	The participants walked in urban or forest environment for 15 min (about 1 km)	1) The trial was conducted in late summer and early autumn of 2014, 2015 and 2017. 2) The trial was conducted in 6 different urban and forest environments with a total of 72 participants. 3) Smoking, drinking alcohol was prohibited, and limited caffeine intake	POMS: D^#^↓ V^#^↑ T-A^#^↓ F^#^↓ C^#^↓ A-H^#^↓ SD method: Comfortable feeling^#^↑ Natural feeling^#^↑ Relaxed feeling^#^↑	lnHF^#^↑ lnLF/lnHF^#^↓ Heart rate^#^↓

*Significant intra-group differences

^#^Significant inter-group differences

*n*, sample size; “↑”, indicators rise; “↓”, indicators decline; “!”, irregular index; *N/A*, no report; “※”, has passed ethical review; “+”, illustrates the grouping method; *ANGII*, Angiotensin II; *A-H*, anger and hostility; *BDI*, Beck depression inventory; *BNP*, Brain natriuretic peptide; *C*, confusion; *Cho*, total cholesterol; *COPD*, chronic obstructive pulmonary disease; *CRP*, C-reactive protein; *D*, depression; *DBP*, diastolic blood pressure; *F*, fatigue; *HRV*, heart rate variability; *HDL*, High density lipoprotein; *IL*, Interleukin; *LDL*, low density lipoprotein; *lnHF*, the natural logarithmic value of the high frequency of heart rate variability; *lnLF*, the natural logarithmic value of the low frequency of heart rate variability; *NK*, Nature killer; *NKT*, Nature killer T; *NRCT*, non-randomized controlled trial; *POMS*, profile of mood states; *RCT*, randomized controlled trial; *SBP*, systolic blood pressure; *SD*, semantic differential; *STAI*, state-trait anxiety inventory; *T-A*, tension and anxiety; *TNF-α*, tumor necrosis factor-α; *V*, vigor; *VAS*, visual analog scale

**Table 3:** Interdisciplinary research (*n* = 3)

Authors (year)	Research design	Participants		Intervention measures	Control measures	Measurements			Outcomes	
		Trial group	Control group			Self-report measures	Physiological measures	Forest inventory		
Takayama (2017) [44]	NRCT (cross-over study)※	1) Male and female participants, the average age was 40.2 years (*n* = 9). 2) Sparse forest environment	1) Male and female participants, the average age was 40.2 years (*n* = 9). 2) Dense forest environment	The trial group was exposed to a sparse forest environment and sat quietly for 15 min, while the control group was exposed to a dense forest environment and sat quietly for 15 min, and then the two groups exchanged environments	1) The trial began in the summer. 2) The trial lasted for 4 days. 3) Alcohol was banned 24 h before the trial and caffeine was banned 12 h before the trial. 4) All subjects did not have a history of cardiovascular disease and psychosis, and did not take medications that could affect their psychology. 5) The interval between the trial in different environments was 10 min	Positive and Negative Affect Schedule: Positive↑ Negative *↓ POMS: D^#^*↓ V↑ T-A↓ F↓ C↓ A-H↓ Perceived Restorativeness Scale: Compatibility scores^#^↑ Restorative Outcome Scale scores↑	N/A	Stand density Stand basal area Species composition Forest photos Hemispherical photograph Forest micrometeorology	Temperature↑ Relative humidity ^#^↑ Wind velocity^#^↓ Radiant heat↑ Illuminance^#^↑ Sound pressure^#^↑	1) Both sparse forest and dense forest had recovery effect on the participants, but the participants evaluated the sparse forest environment more positively. 2) Strengthening forest structure management can improve the healing effect of forest environment on human body
Guan (2017) [45]	RCT	1) Male and female participants, the average age was 22 years (*n* = 20). The environment is birch forest (*Betulaplatyphylla Suk*). 2) Male and female participants, the average age was 21.6 years (*n* = 23). The environment is maple forest (*Acer triflorum*) 3) Male and female participants, the average age was 21.6 years (*n* = 26). The environment is oak forest (*Quercus mongolica*)		The participants were exposed to the forest environment, first taking a tree-measuring course for 20 min, and then enjoying 40 min of private time	1) The trial began in the spring. 2) All participants had no history of cardiovascular disease, allergic symptoms, or mental illness. 3) High-intensity activities, smoking and drinking were prohibited during the trial	Homemade scales: Anxiety caused by employment pressure (birch forest)*↓ Anxiety caused by study interest (maple forest)*↓ Anxiety caused by lesion satisfaction (oak forest)^#^↓	N/A	Height of tree Diameter at breast height Canopy length Canopy cover rate Density	1) The correlation of weight, age and anti-anxiety score was the highest. 2) Forest bathing can promote college students' interest in learning. 3) Overweight young people were better at reducing anxiety. 4) Female participants in the oak forest showed higher levels of anxiety relief than male	
Zhou (2019) [46]	RCT (cross-over study)	Male and female participants, age range 19–23 years (*n* = 24)	Male and female participants, age range 19–23 years (*n* = 19)	Day 1, the trial group was exposed to urban forest park, while the control group was exposed to suburban forest parks. Day 2, the two groups interchanged environments	1) The trial began in the winter. 2) The trial lasted for 2 days	Homemade scales (anti-anxiety score): Finance state*↑ Exam-pass pressure*^#^↓ Campus life*^#^↓ Love affair relationship*↑	N/A	Canopy density Diameter at breast height Plant species	1) The forest richness of suburban forest park is higher than that of urban forest park. 2) Suburban forest park can alleviate interpersonal anxiety in participants more than urban forest parks	

*Significant intra-group differences

^#^Significant inter-group differences

*n*, sample size; “↑”, indicators rise; “↓”, Indicators decline; *N/A*, no report; “※”, has passed ethical review; *A-H*, anger and hostility; *C*, confusion; *D*, depression; *F*, fatigue; *NRCT*, non-randomized controlled trial; *POMS*, profile of mood states; *RCT*, randomized controlled trial; *T*-*A*, tension and anxiety; *V*, vigor

### Evaluative measures

The evaluative measures for the healthcare effect of forest bathing are generally divided into self-reported measures and physiological measures according to different research purposes for choosing the appropriate evaluative measures, both of which can reflect the psychological and physiological stress response of the human body. Self-reported measurement combined with physiological indicators was the largest research method and used a total of 16 studies [19, 20, 22, 23, 25–30, 32–34, 36, 37, 43], accounting for 57% of the total included studies. There are 6 studies each that only use self-reported measurement [21, 35, 40, 44–46] or physiological indicator measurement [24, 31, 38, 39, 41, 42], each accounting for 21.5% of the total number of included studies. Self-reported measurement is widely used because it is simple to measure and easy to conduct quantitative analysis. Currently, internationally accepted self-reported measurement has been applied in the empirical research of forest bathing. Some scholars also use a homemade scale for research [45, 46]. In physiological measures, due to the limitation of the trial environment, blood, urine, or saliva samples that require strict storage time and temperature are generally collected on the spot before and after the forest bathing, or at a place with good medical conditions according to the different testing items. Physiological indicators such as blood pressure, heart rate, pulse, and brain waves are generally measured by portable instruments.

### Physiological response

#### Cardiovascular function and hemodynamic indexes

There were 8 studies [19, 22, 28, 29, 33, 36, 37, 39] involving blood pressure, and systolic blood pressure (SBP) and diastolic blood pressure were significantly reduced in 4 of these studies [19, 22, 33, 37], while only SBP was significantly decreased in 1 study [24], and only SBP was significantly increased in 1 study [15]. There were 4 studies [23, 28, 33, 36] in which pulse was significantly decreased. There were 3 studies [20, 25, 43] involving heart rate, which was significantly decreased in 2 studies [25, 43]. There were 7 studies [20, 25, 29, 33, 34, 38, 43] involving heart rate variability (HRV); the natural logarithmic value of the high frequency (lnHF) of HRV was significantly increased in 4 studies [20, 25, 38, 43], and the natural logarithmic value of the low frequency (lnLF)/lnHF of HRV was significantly decreased in 2 studies [38, 43]. There were 2 studies [32, 39] in which brain natriuretic peptide was significantly decreased. There was 1 study [32] in which Endothelin-1 was significantly decreased.

#### Neuroendocrine indexes

There were 3 studies [23, 27, 31] in which cortisol was significantly decreased. There were 3 studies [22, 27, 28] involving adrenaline, which was significantly decreased in 2 studies [22, 27]. There was 1 study [28] involving norepinephrine and dopamine, which were significantly decreased.

#### Metabolism indexes

There were 2 studies [28, 29] involving triglycerides, which were significantly decreased in 1 study [29]. There was 1 study [28] involving adiponectin, which was significantly increased.

#### Immune and inflammatory indexes

There were 2 studies [27, 41] involving nature killer (NK) cells, which were significantly decreased in 1 study [27]. There was 1 study [27] involving NKT-like cells, which were significantly decreased. There was 1 study [41] involving NK cell activity, which was significantly increased. There were 4 studies [26, 27, 32, 39] involving Interleukin (IL)-6, which was significantly decreased in 2 studies [27, 32]. There were 2 studies [26, 27] involving IL-8, which was significantly decreased. There were 3 studies [26, 32, 39] involving tumor necrosis factor-alpha, which was significantly decreased in 2 studies [26, 39]. There were 3 studies [27, 28, 32] involving C-reactive protein, which was significantly decreased in 1 study [27]. There was 1 study [27] involving IL-1β, Interferon-γ, pulmonary and activation-regulated chemokine, tissue inhibitor of metalloproteinase-1 and surfactant protein D, which were all significantly decreased.

#### Antioxidant indexes

There was 1 study [26] involving glutathione peroxidase, which was significantly increased. There was 1 study [30] involving biological antioxidant potential, which was significantly increased. There was 1 study [42] involving 8-hydroxy-2′deoxyguanosine and hydrogen peroxide, which were significantly decreased. There were 2 studies [32, 39] involving total superoxide dismutase, which was significantly increased in 1 study [32]. There were 2 studies [32, 39] involving malondialdehyde, which was significantly decreased.

#### Electrophysiological indexes

There was 1 study [37] involving electroencephalogram, high alpha brain waves and high beta brain waves, which were significantly increased, and the degree of relaxation of the human body was significantly increased.

### Psychological outcomes

#### Emotional states

There were 14 studies [19, 20, 22, 23, 25, 27, 28, 32, 33, 35, 36, 40, 43, 44] involving the emotional states of humans. Among them, “depression,” “tension-anxiety,” “fatigue,” “confusion,” and “anger-hostility” scores were significantly decreased in 11 studies [19, 20, 25, 27, 28, 32, 33, 35, 40, 43, 44], 13 studies [19, 20, 22, 23, 25, 27, 28, 32, 33, 35, 36, 40, 43], 9 studies [19, 20, 25, 28, 33, 35, 36, 40, 43], 11 studies [19, 20, 22, 25, 28, 32, 33, 35, 36, 40, 43], and 11 studies [19, 20, 22, 25, 27, 32, 33, 35, 36, 40, 43] respectively. There were 10 studies [19, 20, 23, 25, 28, 33, 35, 36, 40, 43] in which the “vigor” score was significantly increased. In addition, 2 studies [35, 44] showed that forest bathing significantly increased positive emotions and decreased negative emotions.

#### Attitudes and feelings towards things

There were 6 studies [20, 22, 23, 25, 37, 43] involving people’s attitudes and feelings towards things; “comfortable,” “relaxed,” and “natural” scores were significantly increased in 5 studies [20, 23, 25, 37, 43], 6 studies [20, 22, 23, 25, 37, 43], and 6 studies [20, 22, 23, 25, 37, 43], respectively.

#### Levels of anxiety and depression

There were 6 studies [30, 33, 36, 37, 45, 46] in which levels of anxiety were significantly decreased. There were 3 studies [29, 30, 34] in which levels of depression were significantly decreased.

#### Degree of physical and psychological recovery

There were 2 studies [21, 26] involving the degree of physical recovery, in which somatic symptoms were significantly decreased. There were 2 studies [35, 44] in which the degree of psychological recovery and mental health were significantly increased.

#### Adaptive behavior

There were 2 studies [29, 34] involving adaptive behavior, and the “self-esteem” score was significantly increased in 1 study [34], and the “health promoting behavior” score was significantly increased in 1 study [29].

### Comprehensive study

**Fig. 2:**
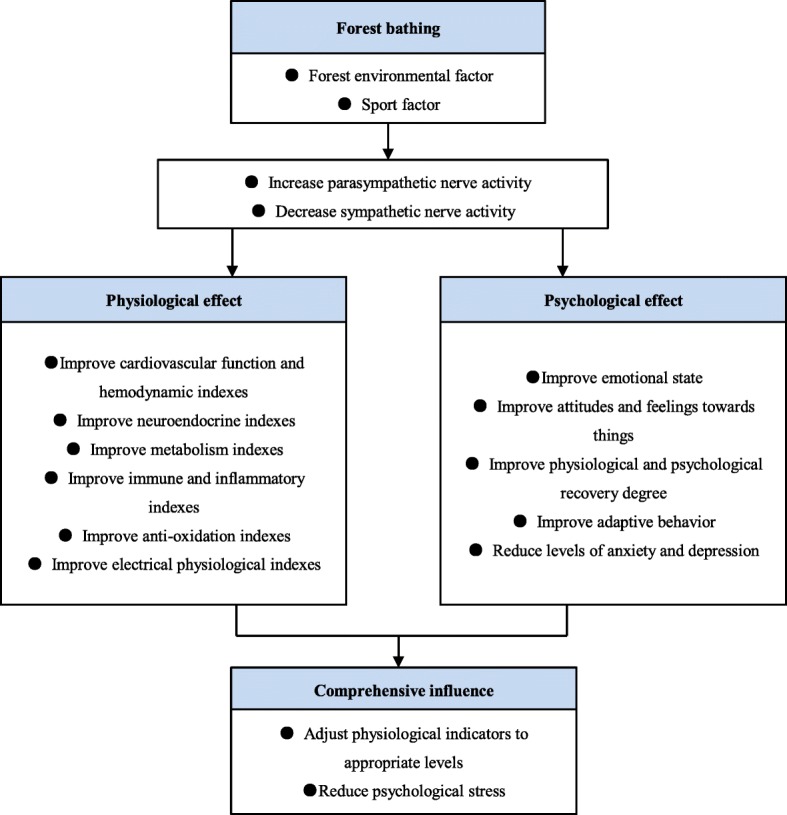
Health benefits of forest bathing

The study of the comprehensive health care effect of forest bathing on the human body is still at the primary stage, and the health care mechanism has not been fully proved. It is general practice to assume that forest bathing has positive effects on the physical or psychological health of a certain group of people (such as cardiovascular disease patients, chronic obstructive pulmonary disease patients, the subhealth population, etc.) and to verify whether this hypothesis is valid. The autonomic nervous system that plays a mediating role in the stress response of various systems has attracted the attention of researchers. Based on the data of the 28 papers included in this study, the lnHF of HRV can reflect parasympathetic activity, and the lnLF/lnHF of HRV, urinary adrenalin and norepinephrine can reflect sympathetic activity [22, 38]. When participants were exposed to walking in the forest environment, the cerebral cortex was in a relaxed state, parasympathetic activity increased (lnHF increased), and sympathetic activity decreased (lnLF/lnHF, urinary adrenalin and norepinephrine decreased) [20, 25]. Cardiovascular function and hemodynamic index, neuroendocrine index, metabolism index, immune and inflammatory index, antioxidation index, and electrical physiological indexes of the human body, emotional state, attitudes and feelings towards things, physiological and psychological recovery degree, and adaptive behavior of the human body were significantly improved. Levels of anxiety and depression were significantly decreased. Song et al. [24] found that high initial values in parameters such as blood pressure and pulse rate in participants were decreased after walking in the forest environment, while participants with lower initial values had the opposite effect. Participants who walked in urban environments did not experience this phenomenon. This indicates that the physiological effect will vary depending on the initial value of the participant, and the forest has a physiological regulation effect close to the appropriate level of the human body, which is not completely caused by the exercise itself. Horiuchi et al. [19] also indicated that the healing effect of forest bathing has nothing to do with the energy expenditure during walking. The health benefits of forest bathing are shown in Fig. 2.

### Quality assessment

**Fig. 3:**
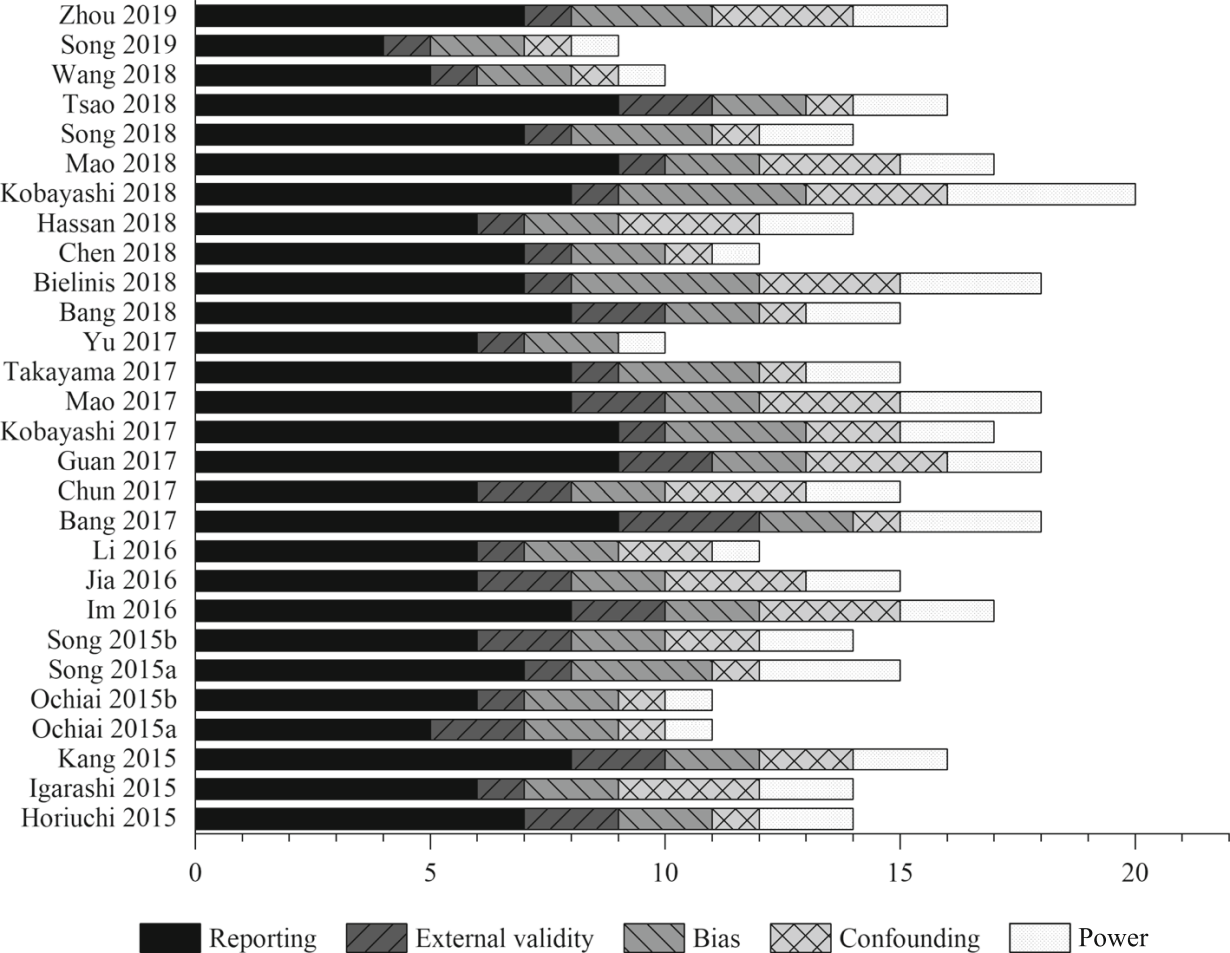
Quality appraisal of included studies using a Downs and Black checklist

**Fig. 4:**
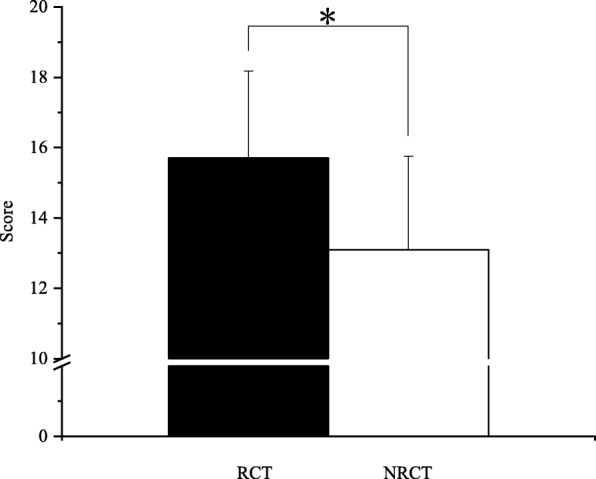
Score of RCT (*n* = 17) and NRCT (*n* = 11), means ± SD, *p < 0.05, one-way analysis of variance (ANOVA)

For methodological quality assessment of papers based on the Downs and Black checklist, of the 28 papers included in the study, 16 [21, 24, 26, 27, 29–32, 34, 35, 38, 39, 41, 44–46] were of high quality and 12 [19, 20, 22, 23, 25, 28, 33, 36, 37, 40, 42, 43] were of low quality (Fig. 3). Among the 16 high-quality papers, there were 12 [21, 24, 26, 27, 30–32, 35, 38, 39, 45, 46] with RCT and 4 [29, 34, 41, 44] with NRCT. The methodological quality of papers using RCT is significantly higher than that of papers using NRCT (*p* < 0.05) (Fig. 4). On the whole, the quality of papers designed with RCT was higher than those with NRCT. In terms of the generation of random sequences, only 1 paper [30] used computer-generated random codes with a low risk of bias. None of the following was mentioned or carried out in the papers: (1) return visit; (2) blind method for intervention practitioners, participants, or data analysts; (3) explain the compliance with the intervention or control measures; and (4) participants who were lost to follow-up were included in the study or carried out the intention-to-treat analysis.

## Discussion

Studies on the health effects of forest environment exposure on the human body are gradually increasing. Currently, there are two main mainstream models. One is the forest bathing model, which advocates subhealthy people and sick people going into the forest for activities which generate a healing effect through forest environmental factors. Forest bathing can regulate blood pressure, reduce blood glucose, regulate endocrine activity, relieve mental disorders, fight cancer, boost immunity, and treat respiratory diseases [3, 47–52]. In recent years, increasing numbers of forest bathing trial studies have been conducted on people with chronic diseases, such as patients with hypertension or high-normal blood pressure [22, 25, 28, 30, 53], chronic obstructive pulmonary disease patients [27], chronic heart failure [32, 39], and chronic stroke [30]. The second is horticultural therapy, which guides sick people into the natural environment and relieves diseases caused mainly by mental stress (excessive tension, panic, insomnia, etc.) through communication with people, making crafts, and gardening activities. Others include pain and sports injuries such as mild hemiplegia, lower body paralysis, and cognitive impairments such as speech disorders, spatial identification disorders, memory disorders, attention disorders, and illogicality [54, 55]. The similarities between forest bathing and horticultural therapy are as follows: (1) They are complementary therapies and cannot replace drugs. (2) They are a healing method to restore the health of the human body through the “five senses experience.” The difference between forest bathing and horticultural therapy are as follows: (1) Their medical categories are different. Forest bathing belongs to the category of preventive medicine, which is mainly aimed at subhealthy people, and the prevention of diseases is its main purpose. Horticultural therapy belongs to the category of rehabilitation medicine, which is mainly aimed at eliminating and reducing dysfunction of the human body, and making up and rebuilding the function of the human body is its main purpose. (2) Their core content is different. The main content of forest bathing is to exercise or meditate in the forest environment, using the forest environmental factors to promote human physical and psychological health. Horticultural therapy is more focused on hand-brain coordination, emphasizing contact with natural things and gaining satisfaction through work. In view of this, different populations should choose appropriate healthcare models. Some scholars [36] combined the 2 healthcare models and achieved very good results.

Based on the data of the 28 papers included in this study, forest bathing has a significant role in promoting human physiology and mental health. Past methods using physiological and self-report measures to distinguish between physiological and psychological research are no longer feasible. The boundaries between the two are becoming increasingly blurred. The mainstream research method in the future will be systematic study of physiological measures combined with self-report measures. For example, in the study of the recovery of physical symptoms or the relief of physiological pain, health effects can be shown by physiological indicators, but self-report measures (such as visual analog scale [21], stress response inventory [26], etc.) can also be used for evidence. Research on the regulation of the human emotional state can use self-reported measures for proof, and physiological measures can also be used for evidence (such as HRV [25, 33, 38], brain wave activity [37], and skin electricity [53]). Although the forest environment has obvious effects on the health of the human body and has achieved certain research results, there are still some problems: (1) Lack of basic theoretical research and multidisciplinary communication. At present, most studies are based on qualitative or quantitative analysis of evidence-based medicine, lacking basic theoretical research of forestry. Medical scholars lack guidance from forestry scholars, relying on subjective or instantaneous forest environmental factor data to determine whether a particular forest has health benefits and environmental factors that lead to increased risk of bias. Forest scholars lack guidance from medical scholars, and the trial participants are mostly healthy young people, most of whom fail to consider ethical issues and measure physiological indicators. Some scholars [45, 46] conduct small sample studies with homemade scales that fail to pass the reliability and validity test, and the evidence is not convincing enough. (2) The risk of bias in the papers is relatively high. Overall, in the 28 papers included in the study, the random sequence generation, the allocation concealment, and the application of blinded methods are important sources of bias. Loss to follow-up, reported adverse events, and intervention-measures or control-measure compliance are the secondary sources of bias. The forest environment is also one of the potential sources of bias.

Interdisciplinary communication between forestry and medicine is an important measure to reduce the bias caused by environmental factors. The forest environment mainly affects human health through “five senses experience,” relying on the synergistic effect between a series of forest environmental factors (such as phytoncide, negative air ions, oxygen, and forest microclimate). These environmental factors have significant seasonal, diurnal, and regional variations. The tree species composition and color and forest density are also important influencing factors and can affect human health, especially mental health. Forest environmental factors in individual studies show that phytoncide with antioxidant and antiseptic enhance immunity function [51, 56]. Air negative ions have the effect of increasing parasympathetic activity, relieving depression, and lowering blood glucose [57–59]. The forest microclimate can improve human thermal comfort and reduce heat stress [60, 61]. A large area of green in the forest can bring a sense of security and calm and significantly reduce anxiety and negative emotions [62]. Comprehensive analysis of the forest environment and dynamic monitoring of key environmental factors are important to judge the potential health benefits of the forest and reveal the healthcare mechanism of forest bathing. This has important guiding significance for the formation of industry standards and the establishment of a forest bathing base.

Reducing the risk of bias is an urgent problem to be solved in medical empirical research of forest bathing, for example, RCT, a method of random sequence generation which should be described in detail. The study of low bias risk should use random number tables, computer software for random number generation, flipping a coin, rolling dice, shuffling cards, or envelopes, etc., rather than odd and even numbers, date of birth, subjective assignment, etc. In forest bathing trials, it is complicated to assign concealment and apply a blind method to participants and personnel. If trial conditions are limited, a cross-over study can be added to reduce the risk of bias, but the length of the washout period should be considered to avoid a carryover effect. Blind methods should be applied to data collectors and outcome assessors to reduce the risk of bias, as conditions permit. Generally, participants are subjectively more inclined to participate in the forest bathing group than the control group. If the guide introduces too much information about the healthcare efficacy of forest bathing, this may give the participants psychological hints, which may increase the risk of bias. Due to the small number of forest bathing test samples and relatively short trial time, the proportion of participants lost to follow-up is small. In case of follow-up loss, the risk of bias can be reduced by estimating the missing data and conducting intention-to-treat analysis. Adverse events such as snake bites, pollen allergies, falls, and bruises were rarely mentioned in the forest bathing study. Adverse events during the trial should be explained in the paper. Compliance with intervention or control measures is also rarely mentioned in forest bathing studies, especially for forest bathing activities greater than one day. Participant compliance with intervention measures such as walking, making crafts, meditating, and taking classes, as well as compliance with restrictions or prohibitions on the use of electronic products, communication, caffeine intake, smoking, and drinking, should be explained.

## Conclusion

Forest bathing activities may significantly improve people’s physical and psychological health. In terms of medical empirical studies on forest bathing, the methodological quality of RCTs is significantly higher than that of NRCTs. In the future, medical empirical studies of forest bathing should reinforce basic studies and interdisciplinary exchange to enhance the methodological quality of papers while decreasing the risk of bias, thereby raising the grade of paper evidence.

## Data Availability

Not applicable

## Appendix

**Table 4:** Search words (subject word and random word)

Intervention	Outcome	Combined terms
1) Forest bathing/	17) Health care/	37) 16 AND 36
2) Forest nature convalescent/	18) Healing/	
3) Forest therapy/	19) Therapy/	
4) *Shinrin-yoku*/	20) Recover/	
5) Forest travel/	21) Vigor/	
6) Forest walking/	22) Spirit/	
7) Forest yoga/	23) Pressure/	
8) Forest/	24) Depression/	
9) Forest meditation/	25) Anxiety/	
10) Forest environment/	26) Brain wave/	
11) Forest areas/	27) Pulse/	
12) Phytoncide/	28) Heart rate/	
13) Negative air ions/	29) Blood pressure/	
14) Negative oxygen ions/	30) Blood glucose/	
15) Oxygen/	31) Saliva/	
16) 1 OR 2 OR 3 OR 4 OR 5 OR 6 OR 7 OR 8 OR 9 OR 10 OR 11 OR 12 OR 13 OR 14 OR 15	32) Inflammatory factor/	
	33) Immune/	
	34) Hormonal readiness/	
	35) Skin conductance/	
	36) 17 OR 18 OR 19 OR 20 OR 21 OR 22 OR 23 OR 24 OR 25 OR 26 OR 27 OR 28 OR 29 OR 30 OR 31 OR 32 OR 33 OR 34 OR 35	

## Abbreviations

HRV: Heart rate variability
IL: Interleukin
lnHF: The natural logarithmic value of the high frequency of heart rate variability
lnLF: The natural logarithmic value of the low frequency of heart rate variability
NK: Nature killer
NRCT: Non-randomized controlled trial
RCT: Randomized controlled trial
SBP: Systolic blood pressure

## References

[1] Xie YM, Liu BY, Piao HY (2006). Exploration on the common characters of sub-healthy people based on clinical epidemiology. Chin J Integr Med..

[2] Zhao H, Xiong WH, Zhao X, Wang LM, Chen JX (2012). Development and evaluation of a traditional chinese medicine syndrome questionnaire for measuring sub-optimal health status in China. J Tradit Chin Med..

[3] Tsunetsugu Y, Park BJ, Miyazaki Y (2010). Trends in research related to “Shinrin-yoku”(taking in the forest atmosphere or forest bathing) in Japan. Environ Health Prev Med..

[4] Kessler RC, Berglund PA, Coulouvrat C, Hajak G, Roth T, Shahly V (2011). Insomnia and the performance of US workers: results from the America insomnia survey. Sleep.

[5] Walsh JK, Coulouvrat C, Hajak G, Lakoma MD, Petukhova M, Roth T (2011). Nighttime insomnia symptoms and perceived health in the America Insomnia Survey (AIS). Sleep.

[6] Fayaz A, Croft P, Langford RM, Donaldson LJ, Jones GT (2016). Prevalence of chronic pain in the UK: a systematic review and meta-analysis of population studies. BMJ open.

[7] Bowler DE, Buyung-Ali LM, Knight TM, Pullin AS (2010). A systematic review of evidence for the added benefits to health of exposure to natural environments. BMC Public Health..

[8] Li Q (2010). Effect of forest bathing trips on human immune function. Environ Health Prev Med..

[9] Oh B, Lee KJ, Zaslawski C, Yeung A, Rosenthal D, Larkey L (2017). Health and well-being benefits of spending time in forests: systematic review. Environ Health Prev Med..

[10] Twohig-Bennett C, Jones A (2018). The health benefits of the great outdoors: a systematic review and meta-analysis of greenspace exposure and health outcomes. Environ Res..

[11] Song C, Ikei H, Miyazaki Y (2016). Physiological effects of nature therapy: a review of the research in Japan. Int J Environ Res Public Health..

[12] Lee I, Choi H, Bang KS, Kim S, Song M, Lee B (2017). Effects of forest therapy on depressive symptoms among adults: a systematic review. Int J Environ Res Public Health..

[13] Hansen MM, Jones R, Tocchini K (2017). Shinrin-yoku (forest bathing) and nature therapy: a state-of-the-art review. Int J Environ Res Public Health..

[14] Furuyashiki A, Tabuchi K, Norikoshi K, Kobayashi T, Oriyama S (2019). A comparative study of the physiological and psychological effects of forest bathing (Shinrin-yoku) on working age people with and without depressive tendencies. Environ Health Prev Med..

[15] Mao GX, Lan XG, Cao YB, Chen ZM, He ZH, Lv YD (2012). Effects of short-term forest bathing on human health in a broad-leaved evergreen forest in Zhejiang Province. China. Biomed Environ Sci..

[16] Shin YK, Kim DJ, Jung-Choi K, Son YJ, Koo JW, Min JA (2013). Differences of psychological effects between meditative and athletic walking in a forest and gymnasium. Scand J Forest Res..

[17] Downs SH, Black N (1998). The feasibility of creating a checklist for the assessment of the methodological quality both of randomised and non-randomised studies of health care interventions. J Epidemiol Community Health..

[18] Moher D, Shamseer L, Clarke M, Ghersi D, Liberati A, Petticrew M (2015). Preferred reporting items for systematic review and meta-analysis protocols (PRISMA-P) 2015 statement. Systematic Reviews..

[19] Horiuchi M, Endo J, Akatsuka S, Hasegawa T, Yamamoto E, Uno T (2015). An effective strategy to reduce blood pressure after forest walking in middle-aged and aged people. J Phys Ther Sci..

[20] Igarashi M, Miwa M, Ikei H, Song C, Takagaki M, Miyazaki Y (2015). Physiological and psychological effects of viewing a kiwifruit (Actinidia deliciosa ‘hayward’) orchard landscape in summer in Japan. Int J Environ Res Public Health..

[21] Kang B, Kim T, Kim MJ, Lee KH, Choi S, Lee DH (2015). Relief of chronic posterior neck pain depending on the type of forest therapy: comparison of the therapeutic effect of forest bathing alone versus forest bathing with exercise. Ann Rehabil Med..

[22] Ochiai H, Ikei H, Song C, Kobayashi M, Takamatsu A, Miura T (2015). Physiological and psychological effects of forest therapy on middle-aged males with high-normal blood pressure. Int J Environ Res Public Health..

[23] Ochiai H, Ikei H, Song C, Kobayashi M, Miura T, Kagawa T, Li Q (2015). Physiological and psychological effects of a forest therapy program on middle-aged females. Int J Environ Res Public Health..

[24] Song C, Ikei H, Miyazaki Y (2015). Elucidation of a physiological adjustment effect in a forest environment: A pilot study. Int J Environ Res Public Health..

[25] Song C, Ikei H, Kobayashi M, Miura T, Taue M, Kagawa T (2015). Effect of forest walking on autonomic nervous system activity in middle-aged hypertensive individuals: A pilot study. Int J Environ Res Public Health..

[26] Im S, Choi H, Jeon YH, Song MK, Kim W, Woo JM (2016). Comparison of effect of two-hour exposure to forest and urban environments on cytokine, anti-oxidant, and stress levels in young adults. Int J Environ Res Public Health..

[27] Jia BB, Yang ZX, Mao GX, Lyu YD, Wen XL, Xu WH (2016). Health effect of forest bathing trip on elderly patients with chronic obstructive pulmonary disease. Biomed Environ Sci..

[28] Li Q, Kobayashi M, Kumeda S, Ochiai T, Miura T, Kagawa T (2016). Effects of forest bathing on cardiovascular and metabolic parameters in middle-aged males. Evid-Based Compl Alt..

[29] Bang KS, Lee I, Kim S, Lim CS, Joh HK, Park BJ (2017). The effects of a campus forest-walking program on undergraduate and graduate students’ physical and psychological health. Int J Environ Res Public Health..

[30] Chun MH, Chang MC, Lee SJ (2017). The effects of forest therapy on depression and anxiety in patients with chronic stroke. Int J Neurosci..

[31] Kobayashi H, Song C, Ikei H, Park BJ, Lee J, Kagawa T (2017). Population-based study on the effect of a forest environment on salivary cortisol concentration. Int J Environ Res Public Health..

[32] Mao G, Cao Y, Wang B, Wang S, Chen Z, Wang J (2017). The salutary influence of forest bathing on elderly patients with chronic heart failure. Int J Environ Res Public Health..

[33] Yu CP, Lin CM, Tsai MJ, Tsai Y, Chen C (2017). Effects of short forest bathing program on autonomic nervous system activity and mood states in middle-aged and elderly individuals. Int J Environ Res Public Health.

[34] Bang KS, Kim S, Song M, Kang KI, Jeong Y (2018). The effects of a health promotion program using urban forests and nursing student mentors on the perceived and psychological health of elementary school children in vulnerable populations. Int J Environ Res Public Health.

[35] Bielinis E, Takayama N, Boiko S, Omelan A, Bielinis L (2018). The effect of winter forest bathing on psychological relaxation of young Polish adults. Urban For Urban Gree..

[36] Chen HT, Yu CP, Lee HY (2018). The effects of forest bathing on stress recovery: evidence from middle-aged females of taiwan. Forests..

[37] Hassan A, Tao J, Li G, Jiang M, Liu A, Jiang Z (2018). Effects of walking in bamboo forest and city environments on brainwave activity in young adults. Evid-Based Compl Alt..

[38] Kobayashi H, Song C, Ikei H, Park BJ, Lee J, Kagawa T (2018). Forest walking affects autonomic nervous activity: a population-based study. Front. Public Health..

[39] Mao GX, Cao YB, Yan Y, Chen ZM, Dong JH, Chen SS (2018). Additive benefits of twice forest bathing trips in elderly patients with chronic heart failure. Biomed Environ Sci..

[40] Song C, Ikei H, Park BJ, Lee J, Kagawa T, Miyazaki Y (2018). Psychological benefits of walking through forest areas. Int J Environ Res Public Health..

[41] Tsao TM, Tsai MJ, Hwang JS, Cheng WF, Wu CF, Chou CK (2018). Health effects of a forest environment on natural killer cells in humans: an observational pilot study. Oncotarget..

[42] Wang DH, Yamada A, Miyanaga M (2018). Changes in urinary hydrogen peroxide and 8-hydroxy-2’-deoxyguanosine levels after a forest walk: a pilot study. Int J Environ Res Public Health..

[43] Song C, Ikei H, Kagawa T, Miyazaki Y (2019). Effects of walking in a forest on young women. Int J Environ Res Public Health..

[44] Takayama N, Fujiwara A, Saito H, Horiuchi M (2017). Management effectiveness of a secondary coniferous forest for landscape appreciation and psychological restoration. Int J Environ Res Public Health..

[45] Guan H, Wei H, He X, Ren Z, An B (2017). The tree-species-specific effect of forest bathing on perceived anxiety alleviation of young-adults in urban forests. Ann For Res..

[46] Zhou C, Yan L, Yu L, Wei H, Guan H, Shang C (2019). Effect of short-term forest bathing in urban parks on perceived anxiety of young-adults: a pilot study in Guiyang, southwest China. Chinese Geogr Sci..

[47] Li Q, Nakadai A, Matsushima H, Miyazaki Y, Krensky AM, Kawada T (2006). Phytoncides (wood essential oils) induce human natural killer cell activity. Immunopharm Immunot..

[48] Li Q, Morimoto K, Nakadai A, Inagaki H, Katsumata M, Shimizu T (2007). Forest bathing enhances human natural killer activity and expression of anti-cancer proteins. Int J Immunopath Ph..

[49] Li Q, Morimoto K, Kobayashi M, Inagaki H, Katsumata M, Hirata Y (2008). A forest bathing trip increases human natural killer activity and expression of anti-cancer proteins in female subjects. Int J Environ Res Public Health..

[50] Li Q, Morimoto K, Kobayashi M, Inagaki H, Katsumata M, Hirata Y (2008). Visiting a forest, but not a city, increases human natural killer activity and expression of anti-cancer proteins. Int J Immunopath Ph..

[51] Li Q, Kobayashi M, Wakayama Y, Inagaki H, Katsumata M, Hirata Y (2009). Effect of phytoncide from trees on human natural killer cell function. Int J Immunopath Pharmacol..

[52] Zheng Q, Yang X (2013). Study and practice of forest-bathing field in Japan. Asian Agr Res..

[53] Mao GX, Cao YB, Lan XG, He ZH, Chen ZM, Wang YZ (2012). Therapeutic effect of forest bathing on human hypertension in the elderly. J Cardiol.

[54] Fletcher L, Hayes SC (2005). Relational frame theory, acceptance and commitment therapy, and a functional analytic definition of mindfulness. J Ration–Emot Cogn –B..

[55] Corazon SS, Stigsdotter UK, Jensen AGC, Nilsson K (2010). Development of the nature-based therapy concept for patients with stress-related illness at the Danish healing forest garden Nacadia. J Ther Hortic..

[56] Abe T, Hisama M, Tanimoto S, Shibayama H, Mihara Y, Nomura M (2008). Antioxidant effects and antimicrobial activites of phytoncide. Biocontrol Sci..

[57] Goel N, Terman M, Terman JS, Macchi MM, Stewart JW (2005). Controlled trial of bright light and negative air ions for chronic depression. Psychol Med..

[58] Bowers B, Flory R, Ametepe J, Staley L, Patrick A, Carrington H (2018). Controlled trial evaluation of exposure duration to negative air ions for the treatment of seasonal affective disorder. Psychiatry Res..

[59] Ohtsuka Y, Yabunaka N, Takayama S (1998). Shinrin-yoku (forest-air bathing and walking) effectively decreases blood glucose levels in diabetic patients. Int J Biometeorol..

[60] De Abreu-Harbich LV, Labaki LC, Matzarakis A (2015). Effect of tree planting design and tree species on human thermal comfort in the tropics. Landscape Urban Plan..

[61] Kong L, Lau KKL, Yuan C, Chen Y, Xu Y, Ren C (2017). Regulation of outdoor thermal comfort by trees in Hong Kong. Sustain Cities and Soc..

[62] Akers A, Barton J, Cossey R, Gainsford P, Griffin M, Micklewright D (2012). Visual color perception in green exercise: Positive effects on mood and perceived exertion. Environ sci technol..

